# Biomarker integration and biosensor technologies enabling AI-driven insights into biological aging

**DOI:** 10.3389/fragi.2025.1703698

**Published:** 2025-11-07

**Authors:** Jared A. Kushner, Mohit Pandey, Sandeep Sonny S. Kohli

**Affiliations:** 1 Diagen AI, Vancouver, BC, Canada; 2 Department of Physics and Astronomy, The University of Victoria, Victoria, VIC, Canada; 3 Department of Biochemistry and Microbiology, The University of Victoria, Victoria, VIC, Canada; 4 The University of British Columbia, Vancouver, BC, Canada; 5 Intensive Care and Internal Medicine, Oakville Trafalgar Hospital, McMaster University, Oakville, ON, Canada

**Keywords:** longevity, biomarkers, C-reactive protein (CRP), interleukin 6 (IL-6), insulin-like growth factor 1 (IGF-1), Growth Differentiation Factor 15 (GDF-15), artificial intelligence, machine learning (ML)

## Abstract

As the global population continues to age, there is an increasing demand for ways to accurately quantify the biological processes underlying aging. Biological age, unlike chronological age, reflects an individual’s physiological state, offering a more accurate measure of health-span and age-related decline. This review focuses on four key biochemical markers - C-Reactive Protein (CRP), Insulin like Growth Factor-1 (IGF-1), Interleukin-6 (IL-6), and Growth Differentiation Factor-15 (GDF-15) – and explores how Artificial Intelligence (AI) and biosensor technologies enhance their measurement and interpretation. AI-driven methods including machine learning, deep learning, and generative models facilitate the interpretation of high dimensional datasets and support the development of widely accessible, data-informed tools for health monitoring and disease risk assessment. This paves the way for a future medical system, enabling more personalized and accessible care, offering deeper, data-driven insights into individual health trajectories, risk profiles, and treatment response. The review additionally highlights the key challenges and future directions for the implementation of AI-driven methods in precision aging frameworks.

## 1 Introduction

While average life expectancy has steadily increased over recent decades, average health-span has remained relatively unchanged, with many individuals experiencing significant health challenges later in life (Garmany and Terzic, 2024; Orrall, 2025). This growing disparity has shifted the focus of the biomedical community towards not only increasing lifespan, but also maximizing the years spent in good health. Biological age, unlike chronological age, reflects the underlying state of the body’s systems and has shown greater utility in predicting disease risk, functional ability, and overall health outcomes (Levine, 2013).

Biological aging is a complex, multi-system process driven by changes at molecular, cellular, and tissue levels. Twelve major categories have been identified as the “hallmarks of aging” which include genomic instability, telomere attrition, epigenetic alterations, loss of proteostasis, disabled macro autophagy, deregulated nutrient sensing, mitochondrial dysfunction, stem cell exhaustion, altered intercellular communication, dysbiosis, and chronic inflammation (López-Otín et al., 2023). Even though the hallmarks of aging represent conceptual frameworks, they reflect underlying disruptions in key biological pathways. These physiological changes, while complex, can be quantified through measurable biomarkers that capture the molecular signatures of age-associated processes.

Aging can be assessed through a wide range of physiological, cognitive, and composite measures that reflect system-level decline. Physical markers such as grip strength, speed, gait, and balance control have been consistently associated with mortality, frailty, and loss of independence (Cesari et al., 2005). Cognitive functions including reaction time and executive control have also been used as a proxy for biological aging, particularly in the context of age-related neurodegeneration (Harada et al., 2013). Cardiovascular markers such as pulse wave velocity and heart rate variability provide insight into vascular aging and cardiovascular disease (CVD) risk (Sessa et al., 2018). Integrated approaches like epigenetic clocks, frailty indices, and AI-driven composite scores combine multiple biological signals into predictive models that estimate biological age and forecast health trajectories (Horvath, 2013; Cole et al., 2017). Together, these diverse biomarker modalities offer comprehensive insights for monitoring the aging process across molecular and functional dimensions.

Among the large range of biomarkers, four have received particular attention due to their reproducibility, accessibility, and links to core aging pathways. These include C-reactive protein (CRP), interleukin 6 (IL-6), insulin like growth factor 1 (IGF-1), and growth differentiation factor 15 (GDF-15). These markers are all involved in key biological processes like inflammation, metabolic regulation, cellular stress, and proliferation; and can be used to estimate biological age and predict related disease risk (Sprott, 2010).

Despite their clinical value, the analysis of biomarker data is often limited by the volume and complexity of information required to draw meaningful conclusions. Advances in artificial intelligence (AI), particularly machine learning (ML) and deep learning (DL), have enabled the efficient analysis of complex, high-dimensional biological data (Topol, 2019). These techniques are now widely used to construct biological age clocks, enhance diagnostic accuracy, and predict health outcomes and disease risk with greater precision. Some recent examples include hematology-based bioclocks, and deep learning algorithms trained on multimodal datasets (Martinez-Romero et al., 2024).

Beyond AI, advances in wearable biosensors now allow for continuous, non-invasive monitoring of physiological signals, providing real time data feedback for health assessment. When integrated with AI, this continuous data stream enables consistent and accurate prediction of disease risk and overall biological age, offering valuable insights into an individual’s current and future health trajectory (Tu et al., 2023). This convergence of biomarkers, biosensors, and AI holds promise for a new era of personalized aging diagnostics and preventative medicine (Topol, 2019; Zhavoronkov et al., 2019).

This review provides a timely synthesis of recent developments linking AI driven analytics and biosensor technology with biochemical biomarkers of aging. While prior reviews have examined these domains independently, this work uniquely integrates them into a unified framework for biological age assessment, highlighting their translational potential for personalized longevity medicine.

## 2 Biomarkers of aging

Biomarkers of aging are measurable indicators that reflect the biological processes underlying aging, offering critical insights into molecular, cellular, and physiological mechanisms. They provide a quantification of age-related decline, offering a measurable distinction between biological age and chronological age. This distinction is critical for assessing healthspan, predicting disease risk, and monitoring the efficacy of therapeutic interventions (Ferrucci et al., 2018).

Age-related biomarkers fall broadly into two categories: biochemical which include a person’s metabolites and circulating proteins; or phenotypical such as one’s gait and grip strength. Biochemical markers are intrinsic to biological processes and are tightly linked to pathways in chronic inflammation, cellular senescence, and metabolic regulation (Franceschi et al., 2018); (Tanaka et al., 2018). Phenotypical biomarkers, in contrast, assess physical function, and serves as a proxy for musculoskeletal integrity and neuromuscular function (Studenski et al., 2014).

An ideal biomarker of aging should be biologically relevant, reproducible, and accessible through non-invasive techniques (Bao et al., 2023).

### 2.1 Applications of biomarkers in aging

Biomarkers of aging are increasingly applied in research and clinical settings to estimate biological age, predict health outcomes, and evaluate intervention efficacy. Composite biomarker-based indices, such as the Dynamic Organism State Indicator (DOSI) and the Physiological Frailty Index, have demonstrated improved performance over chronological age in forecasting all-cause mortality, hospitalization, and frailty (Pyrkov et al., 2021; Blinkouskaya et al., 2021). These models integrate routinely measured blood and physiological variables, providing scalable tools for population-level aging surveillance and personalized risk assessment. In clinical trials, biochemical markers such as GDF-15, IL-6, IGF-1, and CRP are increasingly adopted as surrogate endpoints to track biological responses to interventions including dietary restriction, exercise, and pharmacologic agents (Ridker et al., 2008; Tanaka et al., 2018; Schafer et al., 2020). Longitudinal changes in these markers can reflect alterations in inflammatory burden, metabolic stress, or resilience, offering early signs of intervention effectiveness. As wearable biosensors and remote sampling technologies improve, continuous biomarker monitoring may allow dynamic tracking of individual health, enabling adaptive and precision-based interventions across the aging spectrum. Furthermore, the alignment of biomarkers with known aging pathways, such as inflammation, metabolic regulation, and cellular stress responses, strengthens their utility in assessing the complex multifactorial nature of aging.

**FIGURE 1:**
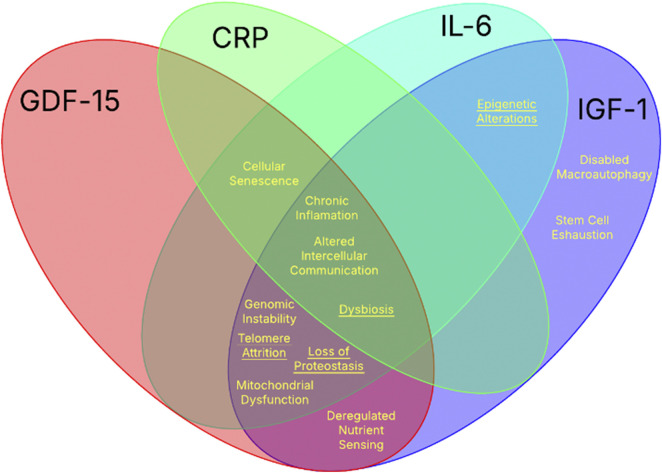
The intersection of the twelve hallmarks of aging with the four key biochemical biomarkers. The Venn diagram illustrates the intersection between the established hallmarks of aging and the biological processes influenced by the four well-studied biochemical aging biomarkers: CRP, IL-6, IGF-1, and GDF-15. Each biomarker contributes to distinct and overlapping hallmarks indicated in yellow (underlined hallmarks signify indirect associations).

Each of the twelve hallmarks of aging are driven by different combinations of biological pathways, each with distinctive associated biochemical markers. The four biochemical markers previously mentioned (GDF-15, IL-6, IGF-1, and CRP) form a strong base for age related diagnostics due to their direct involvement in aging mechanisms. Collectively, these biomarkers provide broad coverage across all twelve hallmarks, with each hallmark intersecting with at least one of the four as seen in Figure 1. This comprehensive overlap underscores their value as an integrated panel for monitoring the complex biology of aging.

### 2.2 Biomarkers in related pathways

Several major biological pathways are involved in the aging process, offering insight into potential targets for biomarkers and therapeutics. The insulin/IGF-1 signaling pathway plays a central role in lifespan modulation and has been extensively linked to longevity across species (Kenyon, 2010). The MAPK/ERK pathway regulates cellular proliferation and responses to oxidative stress, contributing to age-related cellular damage. Similarly, the PI3K/Akt/mTOR pathway is a major regulator of nutrient sensing and growth, and its dysregulation has been associated with age-related metabolic decline and reduced autophagy (Jia et al., 2022); (Yang et al., 2023). Chronic low-grade inflammation, “inflammaging”, is another force of aging, mediated by pro-inflammatory cytokines such as IL-6 and CRP, which are involved in immune signaling and systemic inflammatory responses (Franceschi et al., 2000; Alberro et al., 2021). These inflammatory markers reflect the broader phenomenon of immunosenescence, contributing to increased vulnerability to infections, cancer, and autoimmune diseases. Table 1 highlights the pathways and functions related to each of the four key biochemical markers along with their respective clinical associations. Together, these pathways form the molecular foundation for the understanding of aging and identifying relevant biomarkers.

**TABLE 1:** Summary of key age-associated biomarkers, their molecular binders, associated pathways, biological functions, and clinical relevance.

Biomarker	Pathways	Primary function	Clinical associations
CRP	Classical complement activation, FcγR signaling	Inflammation marker, acute phase reactant	CVD, Diabetes, Cancer, Autoimmune
IL-6	JAK-STAT, Ras-MAPK, PI3K-Akt	Pro-inflammatory cytokine	Frailty, Chronic Inflammation, neurodegeneration
IGF-1	Insulin/IGF-1 signaling, PI3k/Akt, mTOR	Growth Factor, Regulates Metabolism	Longevity, Sarcopenia metabolic syndrome, Cancer
GDF-15	TGF-β, Mitochondrial stress response	Stress-responsive Cytokine, Marker of Mitochondrial Dysfunction	Multimorbidity, Mortality, Cachexia, CVD

### 2.3 The key biochemical markers of aging

#### 2.3.1 C-reactive protein

CRP is a highly conserved member of the pentraxin family critically involved in innate immunity. In its native conformation, CRP has a homopentameric structure composed of five identical non-glycosylated subunits arranged symmetrically around a central pore (Marnell et al., 2005). In addition to the pentameric isoform (pCRP), a monomeric form of CRP (mCRP) is generated through irreversible dissociation at sites of inflammation (Sproston et al., 2018). These two conformations play respective roles in the body’s immune system. pCRP is found primarily circulating in blood (serum) and functions as a pattern recognition molecule facilitating opsonization and the activation of the complement pathway (Thiele et al., 2018). On the other hand, mCRP is typically tissue bound and exhibits potent pro-inflammatory properties through leukocyte recruitment and enhanced cytokine production (Ruiz-Fernández et al., 2021). In response to foreign materials or damaged tissues, circulating CRP (pCRP) can bind phosphocholine on the cell’s surface via calcium-dependent ligand binding leading to the initiation of the classical complement pathway (Williams et al., 2020). The activation of this pathway is mediated through the recruitment of C1q and the direct binding to the Fc region on IgG and IgM antibodies (immunoglobulin) (Marnell et al., 2005). Additionally, CRP can engage with Fcϒ receptors (notably FcϒRI and FcϒRII), promoting immune cell activation and cytokine production (Yang et al., 2007). With the use of high sensitivity assays, circulating CRP levels can be measured for insights into disease risk and inflammatory status. Given its dual role in immune surveillance and inflammation, CRP, particularly high-sensitivity CRP (hsCRP), serves as a robust biomarker of aging. Elevated levels of hsCRP have been consistently linked with age-related diseases such as CVD, frailty and cognitive decline (Ridker et al., 2000).

#### 2.3.2 Insulin-like growth Factor-1

IGF-1 plays a central role in anabolic signaling, cellular growth, and development, making it a key mediator in the aging process. IGF-1 is a key regulator of the insulin/IGF-1 signaling pathway (IIS), binding directly to the IGF-1 receptor (IGF-1R), which modulates lifespan and cellular growth through downstream cascades like the PI3K/Akt/mTOR and MAPK/ERK pathways (Vitale et al., 2019). While IGF-1 has been proven to play a role in lifespan and longevity, its exact role in aging is still hard to understand. While low IGF-1 signaling is linked to increased longevity in model organisms, its role in humans is more complex. Both high and low levels of circulating IGF-1 are associated with increased risk of morbidity and mortality, suggesting a U-shaped relationship (Klinc et al., 2025). Elevated levels have been linked to cancer, while low levels are associated with frailty and CVD (Rahmani et al., 2022). Given IGF-1’s strong association with key aging pathways and diseases, highlights its utility as a biomarker of biological age.

#### 2.3.3 Interleukin-6

IL-6 is a multifunctional pro-inflammatory cytokine that plays a critical role in the body’s immune response and has been continuously implicated in the biology of aging. Produced mainly by immune cells and hepatocytes in the liver, IL-6 initiates the acute-phase response by stimulating the production of CRP (Tanaka et al., 2014). IL-6 levels increase with age (Rea et al., 2018; Puzianowska-Kuźnicka et al., 2016), contributing to a persistent low-grade inflammatory state known as “inflammaging”, which is associated with numerous age-related diseases such as CVD, frailty, sarcopenia and neurodegeneration (Said et al., 2021; Jiménez, 2023). Elevated IL-6 has been consistently linked to physical decline and increased mortality risk in older adults (Ferrucci et al., 2005). In particular, IL-6 impairs muscle regeneration with age by disrupting satellite cell function and promoting catabolic pathways, thereby contributing to sarcopenia and reduced mobility (Muñoz-Cánoves et al., 2013). Furthermore, chronic IL-6 elevation exacerbates oxidative stress and mitochondrial dysfunction, reinforcing the cycle of tissue damage and systemic inflammation (Dai et al., 2014). Given its central role in mediating age-related inflammatory responses and predicting adverse health outcomes, IL-6 is considered a key biomarker of biological aging.

#### 2.3.4 Growth differentiation factor-15

GDF-15 is a stress-responsive cytokine in the transforming growth factor-beta (TGF-β) family. It is widely recognized as a biomarker of mitochondrial dysfunction (Arauna et al., 2020), cellular stress (Schwarz et al., 2023), and tissue injury (García-Esquinas et al., 2022), with levels increasing in response to oxidative metabolic stress. GDF-15 has been strongly associated with aging and age-related pathologies, including CVD, cancer, and frailty, showing consistently elevated levels in older adults (Pence, 2022). A 2025 study showed significantly higher GDF-15 levels in sarcopenic individuals, highlighting its role in muscle loss during aging (Papa et al., 2025). Furthermore, GDF-15 is increasingly recognized as a biomarker intricately associated with epigenetic aging. Epigenome-wide association studies have identified specific CpG methylation sites that are associated with GDF-15 expression, suggesting a potential regulatory relationship between GDF-15 and age-related epigenetic alterations (Moore et al., 2022). Additionally, Recent studies have shown that circulating GDF-15 correlates strongly with several DNA methylation-based clocks, such as GrimAge, PhenoAge, Hannum, and Zhang (Torrens-Mas et al., 2025), thus proving its value as a biomarker of aging. While GDF-15 correlates with multiple epigenetic clocks, its specificity remains questionable due to its elevation in diverse non-age-related acute stress states including cancer, CVD, and renal disease (Pence, 2022; Wan and Fu, 2024).

### 2.4 Additional key biochemical markers

Biological aging is often marked by the dysregulation of cell cycle and tissue maintenance pathways, where specific protein biomarkers such as p21, p16, and Klotho have demonstrated significant relevance. The proteins p21 and p16 are cyclin-dependent kinase inhibitors upregulated during cellular senescence. The accumulation of these kinase inhibitors reflects DNA damage responses and irreversible growth arrest in aging tissues (Sharpless and Sherr, 2015). Notably, p16 is considered one of the most robust markers of senescent cell burden *in vivo* and increases predictably with age across multiple tissues (Liu et al., 2009). Similarly, p21 serves as an early senescence mediator and is closely associated with stress-induced and telomere-dependent senescence pathways (Tao et al., 2024). In contrast, the Klotho protein functions as a longevity hormone, primarily through its role in suppressing oxidative stress and modulating IGF-1 signaling. Klotho expression declines with age and its deficiency leads to accelerated aging phenotypes in mice, whereas its overexpression extends lifespan (Kuro-o et al., 1997; Kurosu et al., 2005). These proteins not only act as mechanistic biomarkers of cellular aging but also represent therapeutic targets in regenerative medicine and senescence-modulating interventions.

There are hundreds of biomarkers to choose from when trying to estimate one’s biological age. For a broader set of aging biomarkers, including their detection methods and applications, see Table 2.

**TABLE 2:** In-depth summary table of known biomarkers of aging with measurement methods and applications (excluding the four key biomarkers).

Marker	Category	Domain	Measurement method(s)	Applications
Heart Rate Variability	Phenotypic	Vascular	ECG, Wearable Heart Rate Sensors	Shaffer and Ginsberg (2017)
Hair colour (gray)	Phenotypic	Integumentary	Visual inspection	Adav and Ng (2023)
Gait	Phenotypic	musculoskeletal	Motion capture, Wearable Sensors	Khera et al. (2023)
Speed	Phenotypic	musculoskeletal	Time-Based Tests	Grande et al. (2019)
Grip strength	Phenotypic	musculoskeletal	Hand Dynamometer	Sayer and Kirkwood (2015)
DNA methylation	biochemical	Genetic	Illumina Arrays, Bisulfite Sequencing	Salameh et al. (2020)
Insulin	biochemical	Endocrine	Blood Assay ELISA	Akintola et al. (2015)
Tumor necrosis Factor (TNF-alpha)	biochemical	Endocrine	ELISA, Multiplexed Cytokine Assays	Lindbergh et al. (2020)
LDL (oxLDL)	Biochemical	Vascular	Blood Test, ELISA	Gradinaru et al. (2015)
Telomere Length	Biochemical	Genetic	qPCR, Southern blot, Flow-FISH	Vaiserma et al. (2021)
Leptin	Biochemical	Endocrine	Blood Test, ELISA	Balaskó et al. (2014)
Adiponectin	Biochemical	Endocrine	Blood Test, ELISA	Li et al. (2021b)
Myeloperoxidase	Biochemical	Immune	Blood Test, Immunoassays	Giovannini et al. (2010)
Chair rise time	Phenotypic	Musculoskeletal	Timed chair stand test, 5-time chair rise test (5CRT)	Meyer et al. (2025)
Balance time	Phenotypic	Musculoskeletal	Timed balance test	Xie et al. (2023)
Reaction time	Phenotypic	Cognitive	Computerized cognitive tests, psychometric tools	Haynes et al. (2017)
Sleep fragmentation	Phenotypic	Cognitive	Actigraphy, polysomnography, wearable trackers	Kaneshwaran et al. (2019)
Pulse wave velocity	Phenotypic	Vascular	Applanation tonometry, oscillometric devices	Marshall et al. (2023)
Frailty index	Phenotypic	Overall	Comprehensive geriatric assessment (CGA), clinical scoring	Blodgett et al. (2024)
6-min walk test	Phenotypic	Musculoskeletal	Timed distance walk	Boxer et al. (2010)
VO2 max	Phenotypic	Vascular	Cardiopulmonary exercise testing (CPET)	Strasser and Burtscher (2018)
Bone mineral density	Phenotypic	Musculoskeletal	Dual-energy X-ray absorptiometry (DEXA), QCT	Consortium et al. (2023)
Skin elasticity	Phenotypic	Integumentary	Cutometer^®^, elastography, indentation-based devices	Runel et al. (2020)
P16	Biochemical	Cell Cycle	qPCR, Western blot, immunohistochemistry	Muss et al. (2020)
P21	Biochemical	Cell Cycle	Western blot, ELISA, immunofluorescence	Yan et al. (2024)
Klotho Protein	Biochemical	Endochrine	ELISA, immunoblotting, blood/urine assays	Hajare et al. (2025)
HbA1C	Biochemical	Endochrine	HPLC, point-of-care devices, immunoassays	Tian et al. (2023)

## 3 Bioclocks

Biological clocks are computational models that estimate an individual’s biological age by analyzing patterns in molecular, physiological, and functional biomarkers. These clocks integrate input from multiple data sources, including DNA methylation profiles, blood-based biomarkers such as CRP, IGF-1, IL-6, and GDF-15, as well as physical and cognitive performance measures. The resulting age estimates often outperform chronological age in predicting morbidity, mortality, and overall health outcomes (Marioni et al., 2015; Hillary et al., 2020). Epigenetic clocks (e.g., Horvath, GrimAge, PhenoAge) have pioneered this field, using machine learning models trained on large datasets of methylation signatures (Horvath, 2013; Lu et al., 2019). A selection of currently available biological age estimation tools, along with their features and data modalities, is summarized in Table 3. Recent approaches to biological clocks incorporate multimodal data sources. These include real-time signals from wearable biosensors such as heart rate variability, physical activity levels, and sweat-based biomarkers. This integration improves both the granularity and temporal resolution of age estimation models.

**TABLE 3:** Summary of available biological age estimation tools and platforms.

Name	Type	Dataset	Description	References
DNAmAge	Code Base	DNA Methylation	First generation epigenetic clock	Horvath (2013)
TruAge	Webtool	DNA Methylation	Commercial epigenetic age testing webtool	TruDiagnostic. (2025)
ClockBase	Webtool/Database	DNA Methylation, blood chemistry	Interactive platform for comparing biological clocks across datasets	Ying et al. (2023)
BioAge	Code base	Clinical Biomarkers	Mortality-based biological age estimator using clinical biomarkers	Kwon and Belsky (2021)
AltumAge	Code base	DNA Methylation	Deep learning-based pan-tissue epigenetic clock implemented in TensorFlow and PyTorch	de Lima Camillo et al. (2022)
DeepMAge	Code base	DNA Methylation	Deep neural network model for predicting biological age from DNA methylation data	Galkin et al. (2021)
MethylNet	Code base	DNA Methylation	Modular deep learning framework for methylation data analysis, including age prediction	Levy et al. (2019)
DnaMethyAge	Code base	DNA Methylation	R package supporting multiple DNA methylation clocks for age prediction	Wang et al. (2024)
Methylclock	Code base	DNA Methylation	Bioconductor package to estimate DNA methylation age using various established clocks	Pelegi-Siso et al. (2021)
Epigeneticclock	Code base	DNA Methylation	Calculates DNA methylation age using Horvath’s method	GitHub, (2013)
Meffonym	Code base	DNA Methylation	R package for DNA methylation-based indices of exposure and phenotype, including age estimation	GitHub, (2025)
TallyAge	Webtool	DNA Methylation	Commercial service for biological age estimation based on DNA methylation patterns obtained from cheek swab samples	Shokhir et al. (2024)

AI plays a critical role in refining these models, especially through deep learning and ensemble techniques that can learn nonlinear relationships between inputs and outcomes. Advanced models can continuously update biological age estimates as new data are collected, enabling adaptive biological clocks that reflect acute changes (e.g., during illness or recovery) and chronic trends (e.g., long-term aging rate). Biosensors provide the necessary infrastructure for continuous data acquisition, allowing biological clocks to function in real time and outside of clinical settings. This convergence enables dynamic health risk stratification, early warning systems for age-related diseases, and longitudinal monitoring of therapeutic interventions, all within a personalized framework (Zhavoronkov et al., 2019; Pierleoni et al., 2021; Wilczok, 2025). Overall, the combination of AI, biosensors, and biomarker data has the potential to revolutionize healthcare and longevity research.

## 4 Wearable biosensors and real-time monitoring

Recent advances in biosensing technologies have enabled continuous, real-time monitoring of age-related biomarkers, marking a significant shift from static measurements to dynamic health tracking. This shift paves the way for personalized healthcare, providing real-time feedback for disease progression and overall health trajectory. Wearable biosensors, especially those capable of non-invasive data collection, are now a critical tool in aging research and personalized health management. Traditional biosensors rely on biochemical detection methods such as electrochemical, optical, or piezoelectric sensing to quantify circulating analytes. These analytes can include a large range of biomarkers; however, this review is focused solely on age-related biomarkers.

Developments in biosensing have expanded the capabilities, allowing for the detection of biomarkers in a wide range of biological mediums such as sweat, saliva, and interstitial fluid. An innovation in biosensing is now able to monitor hsCRP levels in sweat via a wearable wireless patch, therefore bypassing the blood-based assays typically used for inflammatory markers (Tu et al., 2023). Additionally, wearable microneedle patches hold significant promise for the real-time monitoring of biomarkers related to chronic disease and aging by enabling the non-invasive sampling of interstitial fluid (Bao et al., 2024). These patches utilize microneedle arrays that penetrate the stratum corneum without reaching nerve-rich regions, minimizing pain while allowing for sustained sampling (Wang et al., 2021). Other studies have validated the feasibility of using wearable patches to monitor cytokines and protein biomarkers in interstitial fluid or sweat, supporting their application in aging research and chronic disease surveillance (Cha et al., 2025; Wu et al., 2024; Kim et al., 2024). The potential clinical utility of such patches is significant, particularly in early disease detection and management of chronic disease, inflammation, and aging.

## 5 Modeling biological aging with machine learning

ML methods especially with the recent advancements in generative modeling have opened new directions for understanding and forecasting biological aging. Generative models simulate future physiological states, capture latent biological signatures, and enable hypothetical counterfactual analysis. In parallel, predictive ML methods have made significant advances in developing accessible, interpretable, and often clinically actionable biomarkers of aging. Grouping these advances by their application domains provides a clearer picture of how diverse modeling approaches are reshaping aging research.

### 5.1 Brain aging and neurodegeneration

One key frontier is modeling brain aging, particularly using neuroimaging. Conditional generative models trained on cross-sectional 18F-FDG PET scans have been shown to simulate future metabolic topographies in cognitively normal individuals (Choi et al., 2018). These models reveal genotype-specific trajectories; for example, APOE4 carriers show early decline in regions implicated in Alzheimer’s pathology.

Building on this, the SynthBrainGrow model (Zapaishchykova et al., 2025) uses diffusion-based methods to generate realistic MRI scans showing progressive structural changes like cortical thinning and ventricular enlargement. These simulations have been validated against actual follow-up scans, demonstrating their utility in augmenting datasets for neurodegeneration studies.

Conditional normalizing flow-based approaches learn bidirectional mappings between morphology and chronological age (Wilms et al., 2020) through MR images, enabling both brain age predictions and age-conditioned reconstructions of brain anatomy to represent aging trends.

### 5.2 Epigenetic and molecular aging clocks

A second major application is predicting biological age from high-dimensional omics data. Variational autoencoders trained on genome-wide DNA methylation (DNAm) profiles can reconstruct age while discovering biologically interpretable latent features linked to aging (Steyaert et al., 2023). Chromosome-wise autoencoders (Katz et al., 2023) further enhance compression while identifying regulatory CpG sites associated with age acceleration. Cytosine-phosphate-guanine (CpG) sites are regions of DNA where a cytosine nucleotide is bonded to a guanine through a phosphate bond and serves as hotspots for DNA methylation.

Similarly, DL methods have outperformed traditional linear models in age prediction by capturing nonlinear interactions among CpG sites (de Lima Camillo et al., 2022). Due to the non-linear modeling capacity of the neural networks, they offer higher generalizability across tissues and show heightened sensitivity to aging-related diseases, including multiple sclerosis and diabetes.

In male-specific epigenetic clock, a support vector machine trained on Y-chromosome CpG sites created the first sex-specific methylation clock, achieving strong age correlation (Vidaki et al., 2021). Similarly, a cardiovascular health study (CHS)-specific epigenetic clock based on five age-related genes was trained on targeted CpG data, with random forest regression outperforming other models (Fan et al., 2022).

A recent study (Asghari et al., 2025) has further shown that even biomechanical data like upper-extremity function can be used to classify frailty status. By combining movement velocity and muscle co-contraction metrics with LSTM networks, the model effectively distinguished between frail and non-frail older adults.

### 5.3 Simulating aging trajectories and forecasting longevity

Simulating the future course of aging is essential for both basic research and intervention studies. In mice, frailty indices (FIs) have been used to train two random forest models: one predicts chronological age (FRIGHT), while the other (AFFRAID) estimates life expectancy (Schultz et al., 2020). These models have also proven effective in detecting the benefits of longevity interventions, such as drugs or gene modifications, well in advance.

On the human side, the DJIN model (Farrell et al., 2022) represents a stochastic dynamical system that maps how health variables interact over time. Trained on the English Longitudinal Study of Aging, DJIN predicts health trajectories and survival outcomes while uncovering directed relationships among physiological and functional indicators. Unlike traditional survival models, it emphasizes interpretability and interaction structure.

Meanwhile, Sundial (Wu et al., 2025) offers a generative diffusion-based framework that models the molecular aging dynamics (e.g., from transcriptomic, methylation, or other omics data) using a diffusion field without relying on chronological age. It reconstructs personal “aging roadmaps” from cross-sectional omics, enabling the identification of individuals aging faster than average which is a valuable trait for early intervention trials.

### 5.4 Aging biomarkers from lifestyle, microbiome, and Biochemistry

Beyond molecular data, several models have exploited accessible lifestyle, microbial, and biochemical information to predict aging.

A recent study in cerebrovascular disease patients (Fernández-Pérez et al., 2023) used vascular risk factors, organ damage scores, and habits to predict age acceleration. Multilayer perceptrons and elastic net models yielded the best results, showing that such non-invasive data moderately capture epigenetic aging. The integration of microbiome and metabolome data offers another promising avenue. A study on 568 healthy individuals (Seo et al., 2023) trained XGBoost models using 16S rRNA gene sequencing of fecal samples and urine metabolite profiles. Richer microbiome diversity and specific bacterial genera were associated with age, and combining omics improved prediction accuracy, highlighting the utility of non-invasive biomarker fusion.

Another effort built a physiological age model that is independent of chronological age, with biochemical and physiological features such as high-density lipoprotein, pulse wave velocity, and psychological traits (Sun et al., 2021) using statistical ML algorithms. The resulting physiological aging rate correlated strongly with real age and mortality risk and showed ∼30% heritability, suggesting potential for future genetic studies.

Finally, predictive frameworks that integrate gene expression and protein–protein interaction networks have shown that modeling dynamic, weighted interactions lead to better identification of aging-associated genes (Li et al., 2021a). These models outperform static subnetworks and may guide new longevity drug targets.

### 5.5 Identity-preserving face aging and biometric analysis

Generative models also contribute to non-invasive aging tracking using facial features. One approach uses diffusion autoencoders with text-guided embeddings to simulate diverse aging trajectories from a single face image and textual prompts (e.g., “old scientist”, “90s fashion”) (Li et al., 2023). These can be used for health assessments, biometric verification, and age estimation under various lifestyle scenarios. Other methods leverage invertible neural networks to disentangle age from identity, preserving demographic features while generating forward or reverse aged faces (Huang et al., 2021). These models offer robustness and realism often missing in GAN-based face aging methods.

### 5.6 AI-integrated biosensors

The integration of AI into biosensor platforms allows for high-throughput data analysis, enabling the identification of health patterns for biological age estimation. DL models trained on step count and wearable data have already been able to predict morbidity risk with accuracy (Pyrkov et al., 2021), demonstrating the implementation of AI-integrated biosensors. Similarly, AI-enhanced biosensing platforms are increasingly used at point of care to process complex bio-signal data quickly and accurately, enabling personalized interventions (Flynn and Chang, 2024).

Beyond data analysis, AI is now being applied upstream in the design of the biosensors themselves, particularly in the development of molecular binders. AI models, including deep generative architectures and reinforcement learning algorithms, can be used to design high-affinity protein binders for specific analytes such as cytokines, metabolites, or other age-related biomarkers. In the case of CRP, IL-6, GDF-15, and IGF-1, a comprehensive set of validated and computationally predicted binders, along with their sequences and structural representations is detailed in Table 4. These computational approaches enable the *de novo* design of synthetic receptors with tailored binding kinetics, selectivity, and stability, which are critical for the manufacturing of accurate, high precision biosensors. Recent studies have demonstrated that deep learning guided diffusion models and protein design frameworks like RFdiffusion can successfully generate binders for challenging targets (Watson et al., 2023). This reduces the dependence on wet-lab experimental screening and accelerates the creation of custom binders for biosensor manufacturing.

**TABLE 4:** Catalogue of experimental and *in silico* binders for the detection and targeting of key aging biomarkers: CRP, IL-6, IGF-1, GDF-15.

Binder	Type	Structure	Affinity	Source	Pros & Cons
C-Rective Protein					
Phosphocholine	Small molecule	PubChem CID: 1014 PDB: 1B09 (Complex)	*Kd* ≈ 4.8 *μM*	Christopeit et al. (2009)	High specificity for CRP, small size; not ideal for high-throughput sensing due to low signal generation alone
CRP DNA Aptamer	DNA Aptamer	5′-CtA​GTT​CtG​CCt​TAA​TAT​GGt​CGG​tTA​AGC … (48 nt; t = 5-(guanidino)-dU modification)	*Kd* = 6.2 *pM*	Minagawa et al. (2020)	Pros: Ultra-high affinity (picomolar) CRP binder, even higher than original aptamer (53 pM); useful for ultrasensitive CRP detection Cons: Requires modified bases for affinity; potential structural complexity and stability issues *in vivo*; selected *in vitro*, not yet clinically tested
4-C25L22-DQ	Peptide -small molecule	4-C25L22-2-oxo-1,2-dihydroquinoline-8-carboxylic acid (DQ)	*Kd* = 760 *nM*	Yang et al. (2017)	Pros: High affinity, calcium-independent, serum-selective, tunable scaffold, noncanonical CRP site Cons: Undisclosed sequence, complex synthesis, untested *in vivo*, unknown stability
P3	Peptide	VHWDFRQWWQPS	*Kd* = 35 ± 1.2 *nM*	Szot-Karpińska et al. (2023)	Pros: Strong docking, easy to synthesize Cons: No SPR binding, lacks calcium site, unproven in serum
4-C10L17PC6	Peptide-small molecule	4-C10L17-PCh6	*K*d<10 *nM*	Tegler et al. (2011)	Pros: Strong affinity, irreversible binding, highly specific, tunable synthetic design Cons: calcium-dependent, complex synthesis, no *in vivo* or structural data
3-D10L17-PC6	Peptide-small molecule	3- D10L17 (sequence Ac-NAADJEARIKHLAERJKARGPVD CAQJAEQLARAFEAFARAG-CONH2)	Low nanomolar dissociation constant	Fromell et al. (2012)	Pros: Strong ELISA binding, selective for CRP, multivalent, fully synthetic Cons: No KD reported, calcium-dependent, no structural/*in vivo* data
Insulin-like Growth Factor-1					
IGF-1R	Glycoprotein	PDB 7YRR	Kd ≈ 0.16 *nM*	Zhang et al. (2020)	Pros: Natural receptor, very high specificity, can be used in competitive assays Cons: Large size makes it difficult to immobilize on sensors, Expensive to produce
IGFBP-1	Protein	UniProt ID: P08833	K(a) range 1×10^4^ – 9×10^5^ M^-1^ s^-1^ K(d) range 1.5×10^-5^ – 2×10^-4^ s^-1^	Wong et al. (1999)	Pros: High affinity, smaller size enables immobilization, extends IGF-1 half-life Cons: Requires reducing agents to avoid disulfide aggregation, Binds IGF-2 with similar affinity
IGFBP-2	Protein	UniProt ID: P18065	K(a) range 1×10^4^ – 9×10^5^ M^-1^ s^-1^ K(d) range 1.5×10^-5^ – 2×10^-4^ s^-1^	Wong et al. (1999)	Pros: High affinity, smaller size enables immobilization, extends IGF-1 half-life Cons: Requires reducing agents to avoid disulfide aggregation, Binds IGF-2 with similar affinity
IGFBP-3	Protein	UniProt ID: P17936	K(a) range 1×10^4^ – 9×10^5^ M^-1^ s^-1^ K(d) range 1.5×10^-5^ – 2×10^-4^ s^-1^	Wong et al. (1999)	Pros: Most abundant IGF binder in serum, high affinity Cons: Large size, heavily glycosylated, equal affinity to IGF-2
IGFBP-4	Protein	UniProt ID: P22692	K(a) range 1×10^4^ – 9×10^5^ M^-1^ s^-1^ K(d) range 1.5×10^-5^ – 2×10^-4^ s^-1^	Wong et al. (1999)	Pros: high affinity, smaller functional fragments exist, blocks IGF-1R binding site Cons: low serum abundance, binds to IGF-2 with similar affinity
IGFBP-5	Protein	PDB 1H59 (Complex) PDB 1BOE (Domain)	K(a) range 1×10^4^ – 9×10^5^ M^-1^ s^-1^ K(d) range 1.5×10^-5^ – 2×10^-4^ s^-1^	Wong et al. (1999)	Pros: high affinity, mini-IGFBP-5 domain retains full binding site Resistant to proteolysis in serum Cons: moderately binds to IGF-2, multiple disulfides
IGF1-25R	DNA Aptamer	ATC​CGT​CAC​ACC​TGC​TCT​GCA​AGC​ATT​CAT​ATT​GGT​TGG​TGG​AAG​TGG​GGG​GGG​TGG​T GTTGGCTCCCGTAT	High affinity; low nM range Assay LOD: ∼16 ng/mL	Bruno et al. (2016)	Pros: high specificity, small size, rapid binding, reversible denaturation Cons: Kd not explicitly measured, sensitive to nuclease degredation
IGF-1-F1-1	Peptide	PDB 1LB7	*IC*50 = 7.2 ± 3.4 *μM*	Desha et al. (2002)	Pros: small size suitable for biosensor, competitively inhibits IGF-1’s interactions, easy to synthesize Cons: potential cross-reactivity with IGF-2, moderate affinity may require high concentrations for detection
Interleukin-6					
Siltuxi-mab	Antibody	DrugBank ID: DB09036	high-affinity ∼ nM-pM	Marech et al. (2016)	Pros: FDA-approved for Castleman’s disease; neutralizes IL-6 potently Cons: Large biologic (IV infusion); risk of immunogenicity and infections due to immune suppression
Siruku-mab	Antibody	KEGG Entry: D10080	Kd ≈ 0.175 pM	Masjedi et al. (2018)	Pros: High specificity IL-6 neutralization; showed efficacy in rheumatoid arthritis trials Cons: Not approved (development halted); potential safety concerns (infections, neutropenia)
Olokizu-mab	Antibody	KEGG Entry: D12487	Kd ≈ 10 pM	Shaw et al. (2014)	Pros: high affinity, blocks IL-6 at site 3 preventing receptor complex formation Cons: Not yet approved, high cost
SOMA-mer-SL1025	DNA Aptamer	PDB 4N19	Kd = 0.20	Gelinas et al. (2014)	Pros: high affinity, stable chemically modified DNA with slow off-rate Cons: Requires chemical synthesis with modified nucleotides, susceptible to nuclease degradation, not yet used clinically
Clazaki-zumab	Antibody	KEGG Entry: D10312	Kd ≈ 4 pM	Nickerson et al. (2022)	Pros: High affinity, showed efficacy in clinical trials Cons: Development ongoing, safety concerns (as therapeutic)
ZINC2997430	Small molecule	ZINC ID ZINC2997430	Binding energy: −19.15 ± 4.04 kcal/mol	Tran et al. (2022)	Pros: Small molecule, stable in 100 ns MD Cons: No experimental Kd, only *in silico* evidence, off-target specificity untested, not commercially available
Growth Differentiation Factor-15					
GFRAL	Protein	PDB 6WMW (GFRAL receptor w/antibodies) PDB 6Q2J (Complex w/RET)	Kd = 8 nM	Hsu et al. (2017)	Pros: natural high specificity, low immunogenicity Cons: larger size, complex folding, not ideal for biosensors
APT2	Aptamer	5′-AGCAGCACAGAGGTCAGATG-N40-CCTATGCGTGCTACCGTGAA-3′, wherein N is any A, T, G or C	KD = 1.12 nM	Gao et al. (2023)	Pros: Small DNA aptamer, high affinity, chemically stable, modifiable, G-quadruplex fold supports specificity Cons: no peer-reviewed validation, 3D structure unknown

## 6 Challenges

Despite the promise of integrating AI, biosensors, and biomarkers in biological age estimation, several critical challenges must be addressed to ensure accuracy, equity, and real-world applicability. One major concern is algorithmic bias, which arises when training datasets do not represent the diversity of global populations. AI models built on narrow demographic or clinical data can produce skewed or inaccurate results, particularly for underrepresented groups (Char et al., 2018). Developing models that generalize well requires large, diverse datasets that capture a wide range of physiological, genetic, and environmental variability.

Privacy and security are central to the ethical implementation of large-scale data sets containing confidential health information. This requires robust data protection measures and compliance with privacy regulations such as HIPAA and GDPR. Without secure data infrastructures and transparent consent mechanisms, public trust and clinical adaptation may be compromised (Price and Cohen, 2019).

The cost of wearable biosensors along with the computational requirements to run complex AI-based platforms is a potential limiting factor for widespread adoption. Ensuring that the technology is user friendly, accessible, and non-invasive is essential for its utilization in both clinical and everyday settings.

## 7 Future directions

Emerging developments in AI and biosensor integration are poised to further advance the field of aging research. First, the implementation of multimodal aging clocks that synthesize data from molecular, physiological, and behavioral domains is expected to improve the accuracy of biological age estimates. These models could incorporate additional data streams, such as sleep architecture, microbiome composition, or metabolomics, increasing their diagnostic utility.

Integrated AI biosensor frameworks can support real-time monitoring of aging interventions. Lifestyle factors such as exercise, diet, and sleep, along with pharmacological approaches including metformin, rapamycin analogs, or NAD + boosters, can be tracked through changes in the four key biochemical markers. Furthermore, this framework can be implemented directly into healthcare systems allowing physicians to monitor and analyze their patients diagnostics without requiring an in-person appointment.

Biosensors must account for individual variability, including differences in skin pigmentation, hydration levels, and other physiological conditions. Achieving consistent analytical performance requires multi-layer calibration. At the device level, calibration can be done using internal reference signals (such as water and pH) and baseline normalization; at the model-level, calibration is done through algorithms and data selection. Algorithmic bias can be addressed by incorporating diverse cohorts and explicitly reporting subgroup performance metrics.

AI-guided biosensor design is expected to become more sophisticated, incorporating reinforcement learning and structure-aware generative models to design highly specific protein binders for age-related targets. Recent work has already demonstrated the feasibility of using language-based generative models to design protein structures that bind previously undruggable targets (Madani et al., 2023). Additionally, the adoption of federated learning could facilitate model training across decentralized datasets, enabling robust and generalizable predictions while preserving user privacy (Teo et al., 2024). In the commercial sphere, integration into existing consumer health devices could democratize access, providing individuals with feedback on aging trajectories and enabling personalized, non-invasive health interventions.

As these systems mature, interdisciplinary collaborations across geroscience, biomedical engineering, and clinical practice will be critical to validating models, improving interpretability, and ensuring regulatory compliance. These efforts will ultimately shape the development of scalable, ethical, and clinically relevant tools for biological aging assessment.

## 8 Conclusion

Biomarkers such as CRP, IL-6, IGF-1, and GDF-15 provide critical insight into the physiological underpinnings of aging, especially when measured dynamically with modern biosensors and interpreted through AI models. When used in combination, these technologies enable a more refined understanding of health trajectories, allowing researchers and clinicians to track aging in real time and adapt interventions accordingly. However, meaningful application of these tools must address persistent limitations. These include algorithmic bias, limited data diversity, high development costs, and accessibility barriers. Moreover, standardization of methods, cross-disciplinary validation, and patient compliance remain key obstacles to clinical integration. Despite these hurdles, the convergence of biomarkers, biosensors, and AI continues to hold substantial promise. The field is moving steadily toward personalized, data-driven health management, supporting longer and healthier lives through precision aging diagnostics and targeted interventions.
